# Characteristics and Risk Assessment of Microplastics in Water and Mussels Sampled from Cape Town Harbour and Two Oceans Aquarium, South Africa

**DOI:** 10.1007/s00128-023-03737-1

**Published:** 2023-06-07

**Authors:** Conrad Sparks, Nathalie Viljoen, Deen Hill, Jonathan Lassen, Adetunji Awe

**Affiliations:** 1 Department of Conservation and Marine Sciences, Cape Peninsula University of Technology, Cape Town, South Africa; 2 Centre for Sustainable Oceans, Cape Peninsula University of Technology, Cape Town, South Africa; 3 Two Oceans Aquarium, V&A Waterfront, Cape Town, South Africa

**Keywords:** Microplastics, Harbour, Aquarium, Polymers, Risk assessment

## Abstract

The aim of this study was to measure the characteristics and risk assessment of microplastics (MPs) in Cape Town Harbour (CTH) and the Two Oceans Aquarium (TOA) in Cape Town, South Africa from 2018 to 2020. Water and mussel MP samples were analyzed at 3 sites in CTH and TOA, respectively. Microplastics were mainly filamentous, black/grey and 1000–2000 μm in size. A total of 1778 MPs, averaging 7.50 (± 0.6 standard error of the mean, SEM) MPs/unit were recorded. Average MP concentrations were 10.3 ± 1.1 MPs/L in water and 6.27 ± 0.59 MPs/individual or, based on weight, 3.05 ± 1.09 MPs/g soft tissue wet weight in mussels. Average MPs in seawater in CTH (12.08 ± 1.3 SEM MPs/L) was significantly higher (4.61 ± 1.1 MPs/L) than inside the TOA (U = 536, p = 0.04). Various risk assessment calculations indicate that MPs in seawater poses a greater ecological risk than MPs in mussels at the sites sampled.

## Introduction

The increasing production of plastics has resulted in more litter entering the environment, often due to poor waste management practices. This is of particular concern regarding the movement of plastic debris from catchment to coastal areas and the degradation of plastics at both spatial and temporal scales. Plastic debris breaks into smaller particles due to exposure to sunlight (radiant energy), oxygen and mechanical abrasions (Andrady 2011). Larger plastics that subsequently break into smaller plastics between 1 μm and 5 mm in size are classified as secondary microplastics (MPs), with primary microplastics (e.g. nurdles) specifically produced for manufacturing plastic products (GESAMP 2019). It is generally accepted that MPs are components of ocean pollution (Ivar Do Sul and Costa 2014) and determining the prevalence of MPs may be a fundamental step in identifying possible mitigation methods for preventing the occurrence of MPs in the marine environment.

Microplastics have the potential to cause harm to marine organisms (Gall and Thompson 2015; Lusher 2015; Li et al. 2019). Impacts caused by marine animals consuming MPs include mechanical (smothering, hindering digestate mobility and clogging of the digestive tract) and biological effects (hepatic stress, inflammation, impaired movement and slowed growth rates) (Gall and Thompson 2015). Due to its small size, MPs are considered bioavailable to biota throughout the food web and its chemical composition and relatively large surface area makes MPs potentially toxic (Cole et al. 2011). MPs are consumed by various marine organisms, including invertebrates (Cole et al. 2013) and vertebrates (Lusher 2015). Marine mollusks such as mussels are used as biomonitors of environmental pollutants (Bråte et al. 2018). Although an invasive species in South Africa, *Mytilus galloprovincialis* have been used as biomonitors of metals in coastal systems (Sparks et al. 2014) and can be a potential biomonitor of MP pollution (Sparks 2020).

In South Africa, MP concentrations have been recorded in the coastal environment, including coastal waters and sediment (Naidoo et al. 2015; Nel and Froneman 2015; de Villiers 2018; Preston-Whyte et al. 2021) and coastal biota (Naidoo et al. 2015; Nel et al. 2018; Iwalaye et al. 2020; Sparks 2020; Weideman et al. 2020a). According to (Nel et al. 2017), MP concentrations in Cape Town Harbour (CTH) were lower than other harbours in South Africa. The authors argued that this was due to a lack of rivers in Cape Town transporting plastic litter to coastal areas, as evident along the east coast areas of South Africa where plastics are transported to the shore from inland via rivers (Nel et al. 2017).

The Two Oceans Aquarium (TOA) is situated within CTH at the Victoria & Alfred Waterfront, Cape Town, South Africa. The aquarium showcases marine plants and animals commonly found in the warm Indian Ocean and cooler Atlantic Ocean (https://www.aquarium.co.za/). The aquarium extracts seawater from CTH by pumping it from 2 sites, each at a depth of 3 m, to the initial filtration system before being distributed within the aquarium. The aim of this study was to measure the concentrations and characteristics of MPs in CTH and the TOA. The objectives were to: (1) ascertain whether there are differences in MP concentrations and characteristics between CTH (seawater and mussels) and the TOA (seawater); and (2) ascertain the potential ecological risks MPs may pose in CTH and the TOA.

## Materials and Methods

**Fig. 1:**
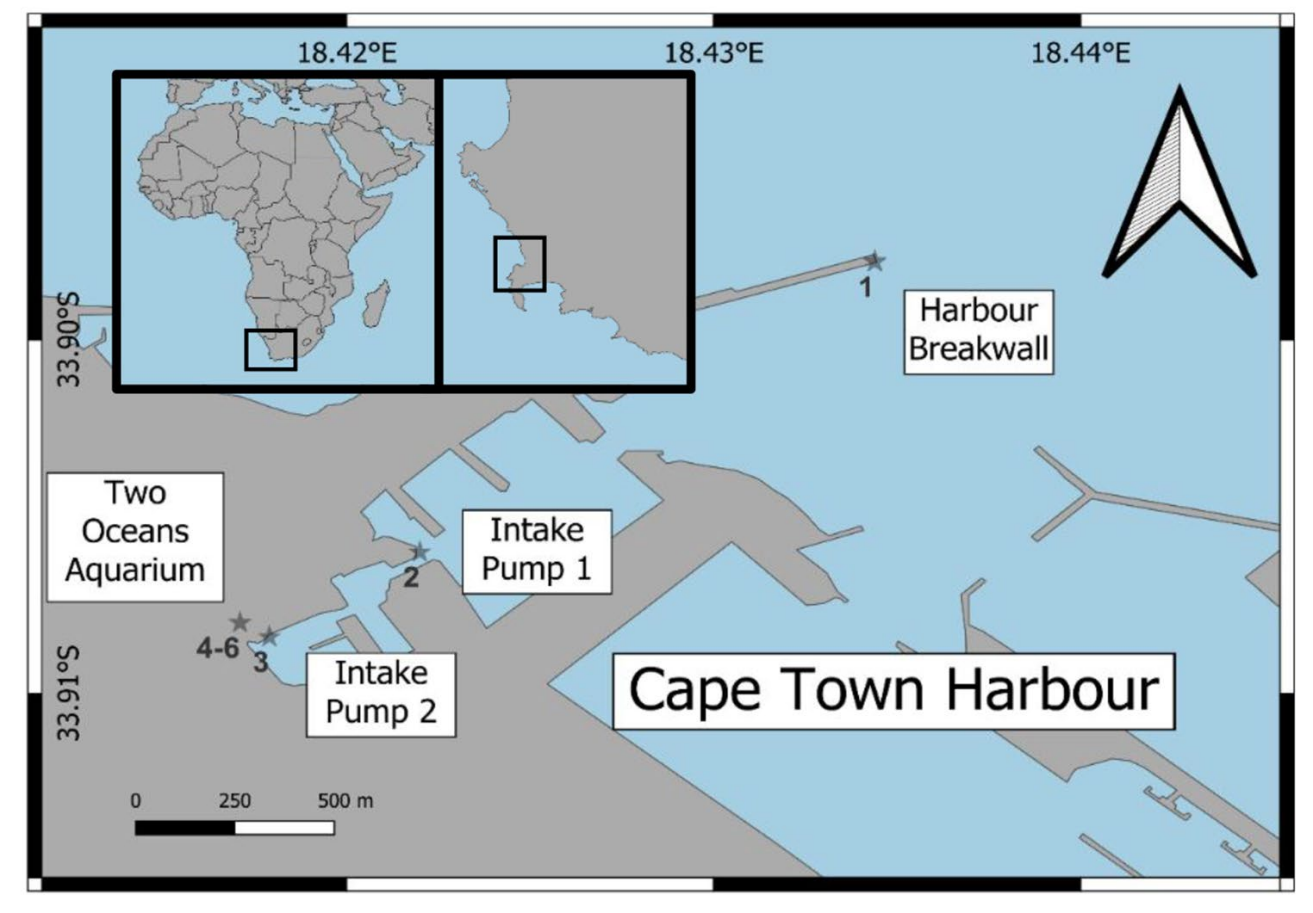
Sampling sites in Cape Town Harbour (CTH) and Two Oceans Aquarium (TOA). Site 1: Harbour Breakwall, Site 2: Intake pump 1, Site 3: Intake pump 2, Sites 4–6: within TOA (see text for explanation)

This study was conducted in the Victoria Basin of Cape Town Harbour (CTH), Cape Town, South Africa (Fig. 1). CTH is situated in Table Bay, a semi-open bay that receives cold nutrient-rich waters from the Benguela Current (Shannon 1985). The current enters the bay from a southerly direction and forms a cyclonic eddy, resulting in a southward flowing counter-current at the surface during winter. Generally, longshore current flow is in a northerly direction (Shannon 1985).

Surface seawater and mussels (*Mytilus galloprovincialis*) were collected from three sites in the Victoria Basin of Cape Town Harbour (Fig. 1) in June 2018, September 2019 and November 2020 at mid-falling tide, as well as seawater samples from three exhibits at the Two Oceans Aquarium (TOA) in September 2019. Site 1 was located at the outer harbour breakwall (33°53’51.3"S 18°26’03.9"E) approximately 1.5 kms northwest from the TOA. Site 2 was located at the TOA intake pump 1 (33°54’21.6"S 18°25’19.3"E), approximately 200 m northeast of TOA. Various commercial, tourist boats and fishing vessels, as well as industrial shipping activities operate near site 2. Site 3 was located at the TOA intake pump 2 (33°54’29.7"S 18°25’05.6"E). Numerous private recreational boats, yachts and sightseeing vessels dock at site 3. Site 4 was the I&J Ocean Exhibit that receives water from intake pump 2 (site 3). It holds 1.6 million L of seawater, has a sandy bottom with a sub-gravel filter, large rock formations and a large diversity of ± 40 marine species. This exhibit is maintained at a temperature of 20 to 24 °C, has a depth of 6 m and displays marine fish diversity of the warm waters of South Africa. Site 5 was in the Predator Exhibit (name subsequently changed to the Save Our Seas Foundation Shark Exhibit) that contains 2 million L of seawater received from intake pump 1 (site 2). The exhibit consists of a sandy bottom (with sub-gravel filtration) with a large rockwork in the centre, is 6 m deep and maintained at 19 to 21 °C. Site 6 was in the Kelp Forest Exhibit that receives water from intake pump 1 (site 2) that contains 800 000 L of seawater. This exhibit is a cold-water display with temperatures varying between 14 and 16 °C and consists of large rockworks, brown kelp (*Ecklonia maxima*) and various fish species.

At all sites, five replicate seawater samples were collected below the surface using 1 L pre-cleaned glass bottles following the sampling protocols of the National Oceanic and Atmospheric Administration (NOAA) guidelines (Masura et al. 2015), with slight modifications. Briefly, each bottle was rinsed three times with site seawater, filled and capped underwater. Seawater samples were transported to the TOA within an hour of collection and stored in a fridge for at least 24 h before being processed at the Cape Peninsula University of Technology Microplastics Laboratory (CPUTML). Twenty mussels (*M. galloprovincialis*) from sites 1–3 were removed by carefully cutting the byssal threads with a 100 mm steel blade and any debris on the outer shell removed. Mussels were immediately placed into labelled bags, stored on ice and later frozen before taken to CPUTML for further processing.

Water and mussel MP samples were processed according to methods adapted from GESAMP (2019). Water samples were filtered through a vacuum pump onto pre-cleaned 20 μm nylon mesh and stored in pre-cleaned closed petri dishes for microscope analyses. Mussels were processed according to the method of Sparks (2020) where mussels were defrosted, lengths measured (mm) and total and soft tissues weighed (g). Soft tissues were digested using a 10% KOH solution, placed in an oven for 24 h at 50 °C, the digestates filtered through a vacuum pump onto a 20 μm nylon mesh and then stored for microscopic analyses. MPs were identified based on shape, colour and size (GESAMP 2019) using a Zeiss Stemi-4 stereoscopic microscope at x20 magnification.

Only polymers sampled in 2020 (only in Cape Town Harbour) were identified spectroscopically using a Perkin Elmer Two ATR-FTIR according to the method of Sparks et al. (2021) as we did not have access to an FTIR in previous years. Spectral wave numbers were set to range from 4000 to 450 cm^− 1^, resolution set at 4 cm^− 1^, data interval set to 1 cm^− 1^ and scans set to 10. Background scans were done before starting scans and the ATR crystal cleaned with propenol between scans. The minimum size limit of MPs analysed was set at 500 μm (n = 155) due to challenges with physically moving MPs to the FTIR and 40% of MPs collected in 2020 were scanned. Polymer identification was done by comparing spectral scans with the ST Japan Library and a Perkin spectral library provided by Perkin Elmer.

Various indices were applied to MPs in seawater and mussels collected in 2020 to assess the potential risks posed by Kabir et al. (2021), with risk categories presented in Table 1. The MP contamination factor (CF) assesses the concentrations of MPs (C_microplastic_) compared to background concentrations

1 $${CF}_{i}= \left(\frac{{C}_{microplastic}}{{C}_{baseline}}\right)$$

where C_baseline_ values selected were the average MPs in mussels reported by (Sparks 2020) (filaments = 6 and fragments = 4 MPs / mussel) and unpublished 2020 data for water in Granger Bay, about 2 km from site 1 and < 1 km from TOA (filaments = 2 and fragments = 0.5 MPs / mussel) (Sparks 2020, unpublished data). We used these values as there are no historic values for the region, the methods were similar and the approach is considered acceptable (Kabir et al. 2021). MP pollution load index (PLI) was calculated for respective MP types

2 $${PLI}_{category}= \sqrt[2]{{{CFr}_{ }}_{category} X {{CFi}_{ }}_{category}}$$

where CFr and CFi were CFs for fragments and filaments, respectively, of a selected category (either site or sample type). The chemical toxicity of polymers were analysed based on the method by (Lithner et al. 2011), where hazard scores are assigned to polymer types to assess the risk of polymers

3 $$H = \sum {P}_{n} \times {S}_{n}$$

where H is the calculated polymer risk index, P_n_ the ratio of a polymer type and S_n_ the polymer hazard score assigned by (Lithner et al. 2011). The pollution ecological risk index (PRI) is calculated as follows

4 $$PRI= \sum H \times {{PLI}_{category}}_{ }$$

where PRI indicates the ecological hazards posed by polymers, based associations between pollution loads (PLI) and the polymer risk index (H).

**Table 1:** Risk categories of indices for microplastic contamination (Kabir et al. 2021) in Cape Town Harbour and the Two Oceans Aquarium in 2020

Risk Category:	Low (I)	Moderate (II)	High (III)	Very High (IV)	Dangerous (V)
Contamination Factor (CF)	< 1	1–3	3–6	> 6	
Pollution Load Index (PLI)	< 1	1–3	3–4	4–5	> 5
Polymer Risk Index (H)	< 10	10–100	101–1000	1000–10,000	> 10,000
Pollution Risk Index (PRI)	< 150	150–300	300–600	600–1200	> 1200

Quality controls were followed both in the field and lab according to accepted protocols (GESAMP 2019). In the field, glass containers were pre-cleaned with reverse osmosis (RO) water and the use of plastic items were kept to a minimum. In the lab, the same clothing, cotton lab coats and gloves were worn, with all glassware and equipment rinsed three times with RO water. All glassware, equipment and containers were kept covered with aluminum foil to prevent air-borne contamination. The doors of the lab were kept closed and empty petri dishes placed next to workbenches to report any airborne contamination. No MP particles were reported for airborne contamination. Three blanks were processed when doing filtrations for both water and mussel samples and 6 MPs were recorded for the duration of all lab analyses. We considered these values negligible and did not factor this in MP concentration calculations. Extraction efficiencies were done for the 2020 samples only (but the same process was followed as for 2018 and 2019) and 90% efficiency recorded for MP fragments 500–1000 μm in size and 85% efficiency for MP filaments 500–1000 μm in size.

All statistical analysis was conducted using SPSS V28. Assumptions of normality for seawater and mussel samples were tested using the Shapiro-Wilk tests and tests for homogeneity of variance conducted using the Levene’s statistical test. Assumptions of normality and equal variances for mussel and seawater samples were not met (even after log transformations) and non-parametric tests performed using the Mann-Whitney U test between 2 groups and the Kruskal-Wallis (KW) test for multiple groups. Significance was set at p < 0.05 and variability of data expressed as standard error of the mean (SEM).

## Results and Discussion

A total of 243 samples were collected from 6 sites between 2018 and 2020 with MPs recorded in 94% of samples processed. Of samples analyzed, 63.5% of MPs were found in mussels and 36.5% in water samples. A total of 1778 MPs were recorded from all samples processed, an average of 7.50 (± 0.6 SEM) MPs/unit in CTH and 4.60 (± 1.1) MPs/unit in TOA.

**Fig. 2:**
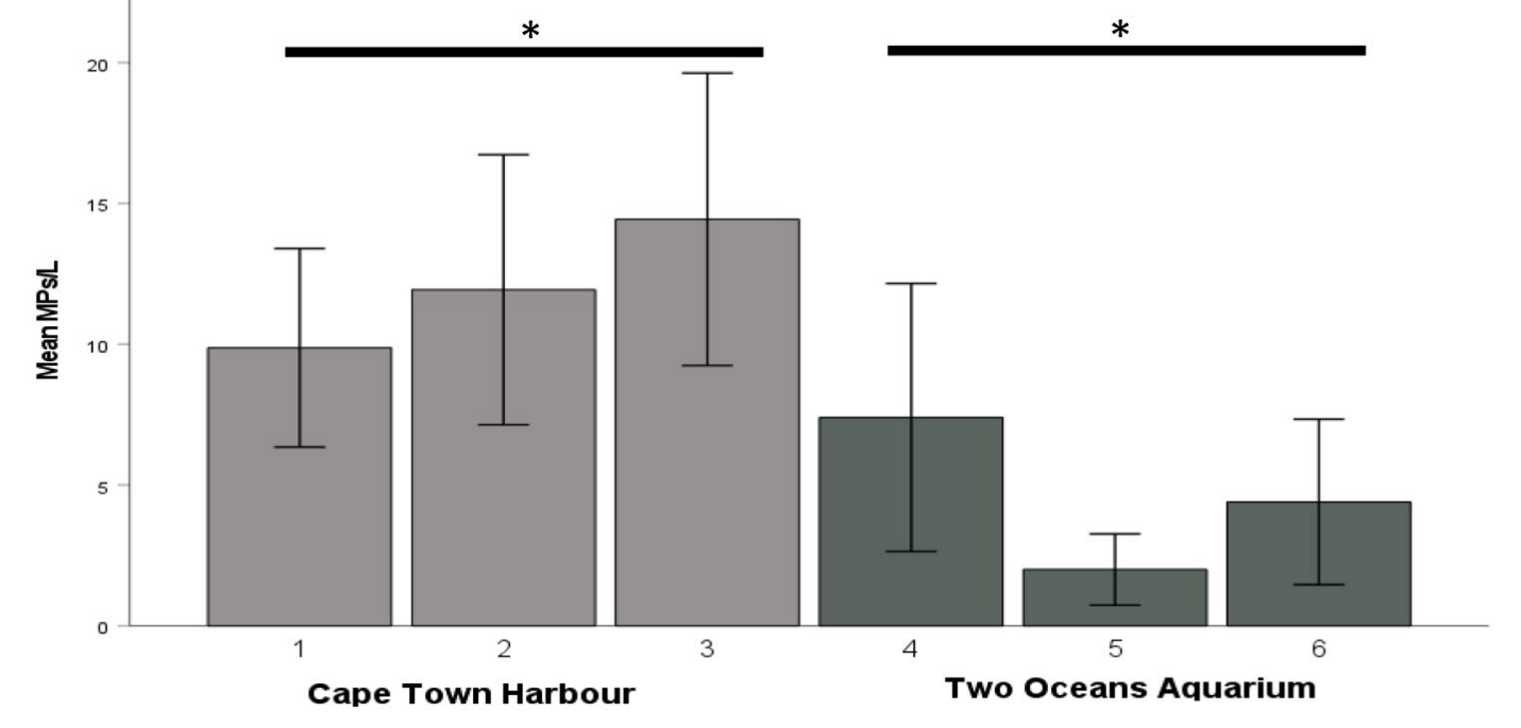
Mean water MP concentrations at sites in Cape Town Harbour (CTH) (sites 1-3) and Two Oceans Aquarium (TOA) (sites 4-6). Error bars = standard error of the mean. * Indicates significant differences between CTH and TOA

Average MPs in water from all sites was 10.3 ± 1.1 MPs/L and MPs in water from CTH (12.08 ± 1.3 SEM MPs/L) were significantly higher than TOA (4.61 ± 1.1 MPs/L, U = 536, p = 0.04) (Fig. 2). MPs from CTH water samples were highest at site 3 (14.44 ± 2.6 MPs/L) (pump 2) adjacent to TOA and lowest at site 1 (9.88 ± 1.78 MPs/L), the edge of a breakwater of the harbour, about 1.5 km northeast of TOA. Within TOA, water MP concentrations were highest at site 4 (Oceans Exhibit) (7.43 ± 2.33 MPs/L) and lowest at site 5 (Predator Exhibit) (2.01 ± 0.6 MPs/L). There were no significant differences in MP water samples between the 3 sites sampled in CTH or TOA (p > 0.05).

Compared to other harbours in South Africa, MPs reported here are by orders of magnitude higher than other ports. Durban harbour is one of the busiest and largest ports in South Africa (Preston-Whyte et al. 2021) and previous MP concentrations recorded in Durban harbour were 0.01 MPs/L (Preston-Whyte et al. 2021), 1.20 ± 0.13 MPs/L (Nel et al. 2017) and 0.007 ± 0.012 MPs/L (Naidoo et al. 2015), which were orders of magnitude lower than recorded in CTH. However, it should be noted that the different sampling protocols and laboratory analyses may affect the final reporting of MPs data, and these comparisons should be made with caution.

Water samples collected in the TOA were obviously lower than CTH, as there are numerous processes within TOA to filter and purify seawater. However, the high percentage filaments at site 4 requires further investigation (see Fig. 2) as site 4 received CTH water from site 3 and was the highest MPs recorded of the 3 sites sampled inside TOA. The results are also promising from the view that the water quality within TOA is relatively good in terms of the low number of MPs (monitoring MP concentrations within TOA are not regularly done). However, when compared to the Seattle Aquarium (USA), mean water MP concentrations are higher than the Seattle Aquarium, where the mean water MP concentration was 0.24 ± 0.004 MPs/L and ranged from 0.00 to 0.64 sampled for the period January 2019 to January 2021 (Harris et al. 2021).

**Fig. 3:**
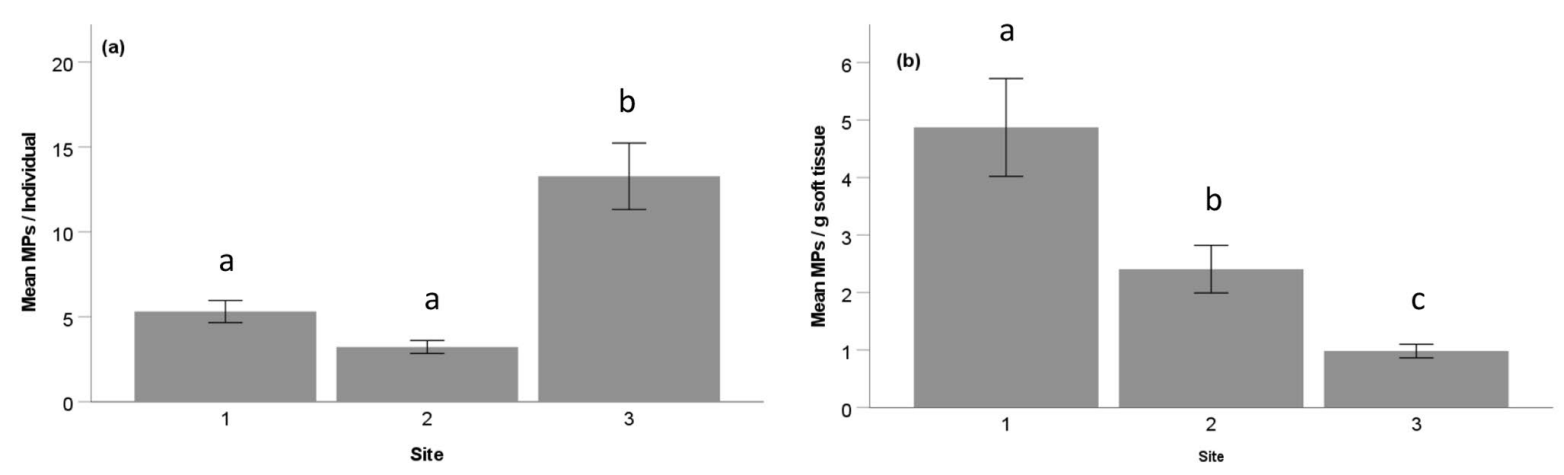
Microplastic abundances (**a**: particles per individual and **b**: particles per gram soft tissue wet weight) in mussels from Cape Town Harbour, South Africa. Different letters indicate significant differences between groups for respective graphs

Mussels had an average of 6.27 (± 0.59) MPs/individual and 3.05 (± 1.09) MPs/g soft tissue wet weight (Fig. 3). Mussel MP concentrations in CTH were higher than previously recorded in Cape Town (4.27 MPs/individual and 2.33 MP/g soft tissue weight) (Sparks 2020). Based on MPs per individual (Fig. 3a), the significantly higher (KW, H = 14.23, p = 0.01) MP concentrations recorded at site 3 is indicative of a lack of circulation within CTH. Bodies of coastal water with intensive levels of anthropogenic activities such as harbours in urban centres are known to be contaminated with litter and MPs (Sundar et al. 2020). The increased anthropogenic inputs and poor water quality are potential factors for “Trojan horse” effects that influences contamination of urbanized coastal water bodies such as harbours (Hildebrandt et al. 2021). This scenario was particularly evident in recent years in CTH when major fish kills occurred due to suspected poor water quality and low circulation during summer in Cape Town. Based on weight (Fig. 3b), mussel MP concentrations were significantly highest at site 1 (KW, H = 12.1, p = 0.02) and was most likely due to smaller mussels processed at site 1. Mean mussel sizes (mm) were 25 (± 2.1) mm at site 1, 25 (± 2.5) mm at site 2 and 67 (± 2.2) mm at site 3.

**Fig. 4:**
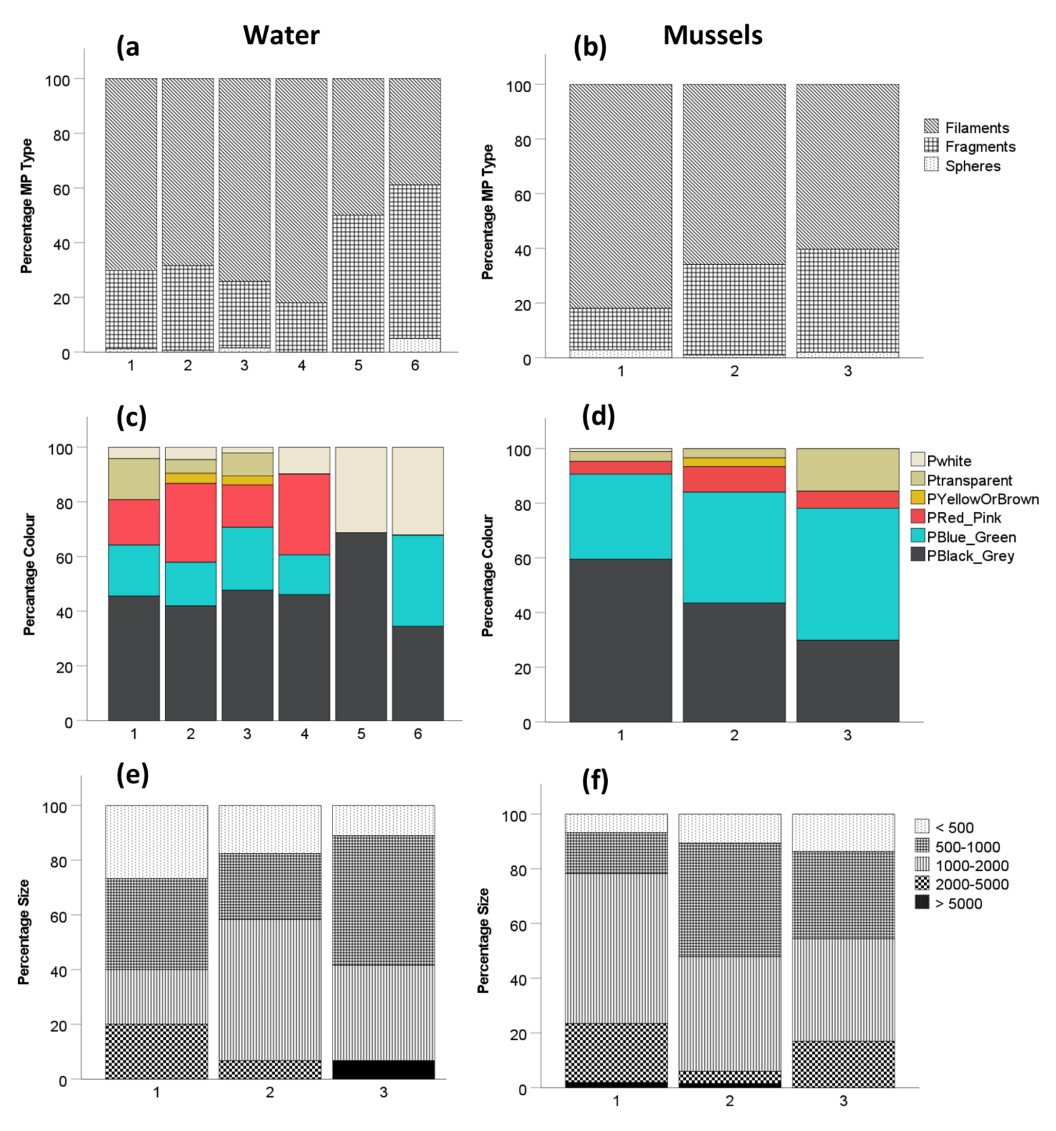
Microplastic type (**a** and **b**), colours (**c** and **d**) and sizes (µm) (**e** and **f**) in water ((**a**, **c** and **e**) and mussels (**b**, **d** and **f**) sampled from Cape Town Harbour (sites 1–3) and Two Oceans Aquarium (sites 4–6), South Africa. (Note: no size analyses of MP water samples were done at sites 4–6 – Fig. 4e)

MP characteristics were not similar in samples at CTH and TOA (Fig. 4). Filamentous MPs (70%) were the most common types sampled across all sites, followed by fragments (28%) and spheres (2%) (Fig. 4a and b). Filaments were predominant in water samples at site 4 (Fig. 4a) and mussels (82%) at site 1 (Fig. 4b). For all sites combined, black/grey were the most frequent colours recorded in MP water samples (46%), followed by blue/green (18%) and red/pink (17%), respectively (Fig. 4c). Black/grey MPs occurred most frequently in water samples at site 5 (68%) (Fig. 4c). For all sites combined, black/grey were the most common MPs found in mussels (49%), followed by blue/green MPs (37%) (Fig. 4d). Highest black/grey MP concentrations were recorded in mussels at site 1 (60%) and blue/green in mussels at site 3 (48%). MPs between 1000 and 2000 µm were the most common sizes in water, 52% at site 2 (Fig. 4e) and 48% in mussels at site 1 (Fig. 4f).

Descriptions of MP characteristics are important for baseline investigations as the type, colour and sizes of MPs have the potential to have an impact on coastal biota, especially in enclosed areas such as harbours. Filamentous MPs are the most common type of MPs recorded in coastal waters and mussels (Qu et al. 2018) and are potentially more toxic than other MPs in the environment (Jemec et al. 2016). We report similar results here and hence note that MPs in mussels may be bioavailable to their predators in the immediate area (e.g. starfish and seagulls). The filaments, colour (black/grey) and size (500–2000 μm) of MPs reported ﻿ in mussels from this study are similar to that reported in Cape Town (Sparks 2020) and elsewhere (Zhao et al. 2014; Qu et al. 2018) and shows that there is a need to investigate the rates of uptake and effects of these MPs in southern Africa. Previous studies from elsewhere indicated that MPs in coastal waters are ingested by mussels (Brown et al. 2008) and MPs smaller than 1000 μm are highly toxic to invertebrates (Guzzetti et al. 2018).

Single-use plastics such as packaging materials, fishing gear and plastic products are the main types of plastic litter (and hence potential sources of MP pollution) in South Africa (Ryan and Moloney 1990). These plastics may enter CTH by means of urban and stormwater run-off from Cape Town during the rainy season in winter ((Weideman et al. 2020b) and offshore winds blow urban litter from land to the sea in summer (Ryan 2020). Other potential sources of MP pollution in CTH may include municipal sewage discharged and stormwater systems into Table Bay (Petrik et al. 2017) as well as maritime and fishing activity. Plastic pollution reduces the aesthetic value of tourist hotspots such as the V&A Waterfront and has the potential to damage vessels where discarded ropes, nets and packing bands may become entangled in propellers (Andrady 2011). Plastic and microplastic pollution poses a threat to animals such as seals, birds and invertebrates which frequent and reside in CTH (Gardner et al. 2021), as they may become entangled in discarded rope and packing bands or ingest plastic materials (Shaughnessy 1980; Gall and Thompson 2015).

**Fig. 5:**
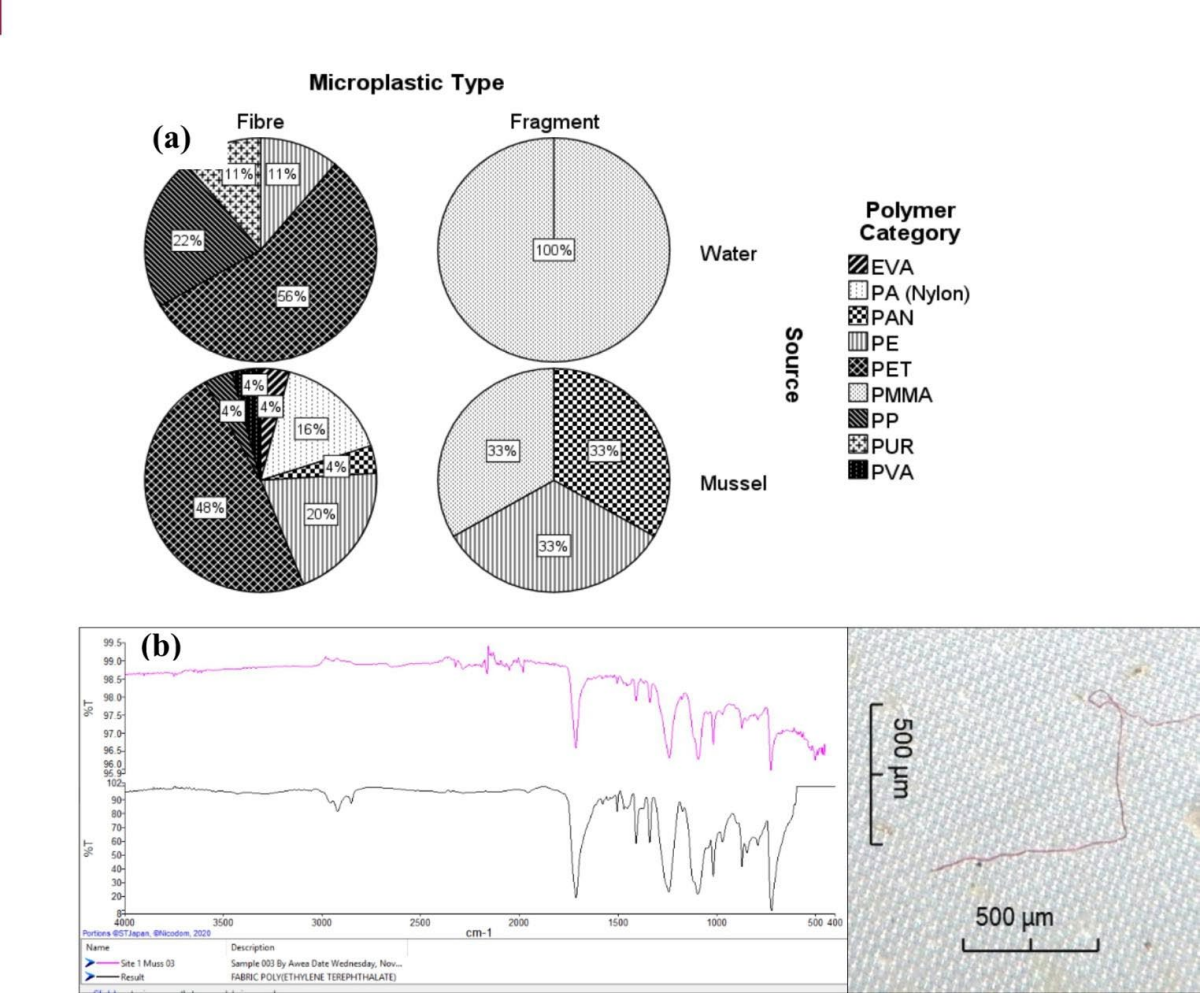
(**a**) Microplastic polymer types sampled at sites 1–3 (Cape Town Harbour). Polymer abbreviations: ethylene vinyl acetate (EVA), polyamide (PA), polyacrylonitrile (PAN), polyethylene (PE), polyethylene terephthalate (PET), polymethyl methacrylate (PMMA), polypropylene (PP), polyurethane (PUR), polyvinyl acetate (PVA) and (**b**) and example of an FTIR scan and associated picture of the MP fibre analysed

A total of 62 MPs were scanned (47% of MPs recorded) for polymer identification, which comprised 57 (92%) filaments, 4 fragments (6%) and 1 foam (2%) MPs (Fig. 5). Of the MPs processed, we analysed 73% from mussels and 27% from water samples and of all MPs processed, 62% were synthetic (polymers) and 38% not polymers (e.g. cotton, rayon and cellulose). For all sites combined, 45% of polymers were PET and 18% PE. In mussels, filaments were 48% PET and 20% PE, and fragments were 50% PE and 50% PMMA. In water, filaments were 56% PET and 22% PP, and fragments were 100% PMMA (Fig. 5a). Figure 5b shows the FTIR scan of a red filament found in a mussel at site 1.

Polymer identification is becoming a prerequisite measurement to report on when conducting research on MPs in the environment. The most common polymers in the marine environment are PE, PP, PS, PA, PET and PVC (Andrady 2011). In our study, we recorded mainly PET and PE, which could have derived from numerous sources, as discussed previously. An interesting result from FTIR analyses was that 62% of MPs processed were categorized as polymers which means that 38% were not synthetic MPs. The effects of MP uptake are still poorly understood (ie, whether synthetic or natural) but includes damage to guts and death due to starvation as a result of animals feeling satiated by the presence of MPs in their gut (Ma et al. 2020).

**Fig. 6:**
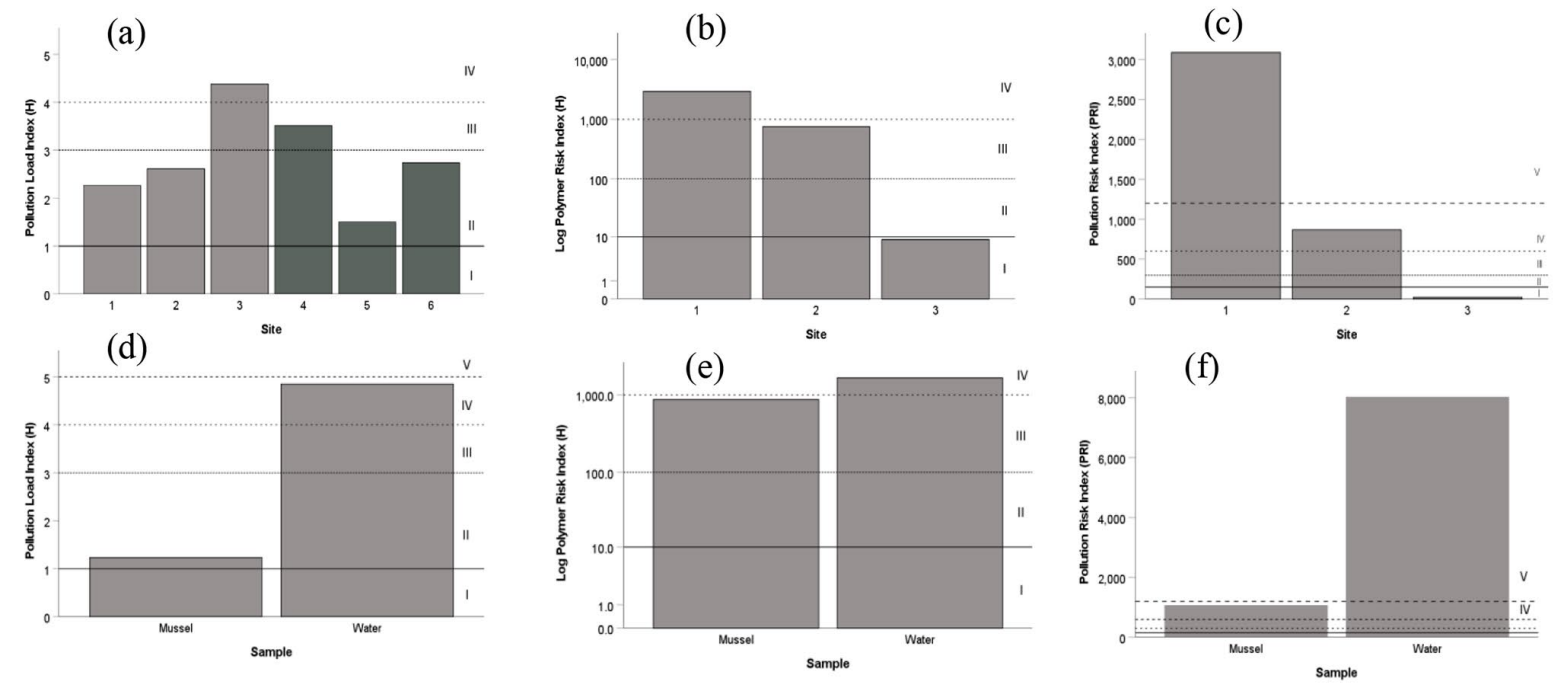
Risk assessment of sites (**a-c**) and sample type (**d-f**) based on Pollution Load Index (**a** and **d**), log Polymer Risk Index (**b** and **e**) and Pollution Risk Index (c and f) of microplastics sampled in water and mussels at sites 1–3 in Cape Town harbour (grey) and sites 4 to 6 (Two Oceans Aquarium, dark grey) as well was mussels and water in 2020 only at sites 1 to 3. See Table 1 for categories of indices (I – IV). Note the log scale for the Polymer Risk Index

Risk assessment indicated that polymers in water and mussels in CTH and TOA poses environmental risks (Fig. 6). Based on sites, the highest pollution load was at intake pump 2 (CTH site 3) (Fig. 6a). The PLI value of IV is categorized as very high. The highest PLI within TOA was at site 4 (I&J Ocean Exhibit). Given the large size of the exhibit (1.6 million L water), sandy bottom and enclosed structure, MPs in the exhibit may be accumulating over time. Site 4 had the highest concentrations of MPs in TOA (see Fig. 2) and sources of MPs may be from the intake pump 2 (site 3) and from MPs in feeds being given to animals in the system. Although the sources of feed are varied, it includes sardines (*Sardinops sagax*) and hake (*Merluccius spp.*). MPs were reported in guts of sardines sampled from the south and west coast of South Africa (Bakir et al. 2020) and in hake guts from the south coast of South Africa (Sparks and Immelman 2020). Further investigation of potential sources of MPs in feeds used at the TOA is advised. The Polymer Risk Index (H) (Fig. 6b) and Pollution Risk Index (PRI) (Fig. 6c) followed similar trends, highest at site 1, decreasing to site 3. At site 1, H was category IV (very high risk) and PRI at category V (very dangerous). The high risk values recorded at site 1 requires further investigation as the site was the furthermost of all sampled in CTH and is downstream from a sewage outfall pipe (Petrik et al. 2017), which are known to be a sources of MPs (Mahon et al. 2017). All risk indices of water samples were higher than mussels (Fig. 6e-f), suggesting that organisms (such as mussels) have the potential to reject and eject MPs (Graham et al. 2019), thereby reducing the potential effects of ingested MPs. However, the mechanisms of this for organisms in South Africa requires further investigation.

To our knowledge, the current investigation is the first to monitor the abundance and types of MPs in an aquarium in South Africa. Aquariums are popular tourist destinations, and the TOA is located in the most popular tourist destination of Cape Town, the V&A Waterfront. The aquarium displays an array of marine animals, providing important services to educate visitors (including children). The TOA also plays a significant role in marine conservation, providing rehabilitation facilities and services for marine animals such as sea turtles (Ryan et al. 2016). Even though MPs (filaments) were highest at site 4 (Oceans Exhibit) and the source of MPs considered to be from site 3 (intake pump 2), other potential sources of MPs in TOA require further investigation. This could include equipment and gear used to clean areas, type of clothing worn by staff, MPs from the atmosphere (from net shade cloth), sand that is constantly replaced, whole small fish fed to animals in the aquarium and the municipal water used in the TOA. Knowledge about the sources of MP in TOA will enable mitigation measures to be put in place to reduce the potential impacts MPs may have on animals in TOA.

## Conclusion

In this study we described the concentrations, characteristics and ecological risks of MPs in Cape Town Harbour and the Two Oceans Aquarium in Cape Town, South Africa. The MP concentrations recorded were higher than previous studies in the region (Sparks 2020) and provides a first account of ecological risk assessment of MPs in Table Bay. MPs were mainly filamentous, black/grey and between 0.5 and 2 mm in size. The main polymer type of filaments were PET and fragments, PMMA. The characteristics of MP polymers in water and mussels were not the same and risk assessments indicated that polymers in water posed greater risks than in mussels. The high risk assessment values reported suggests that filamentous MPs in Cape Town coastal waters have the potential to negatively affect organisms in enclosed/confined areas (such as harbours and aquaria). Hence, the results of the research provide motivation for MPs to become part of coastal monitoring programmes in the future.

## Data Availability

The datasets generated and/or analyzed during the current study are available from the corresponding author on reasonable request.
